# Computer vision syndrome with academic burnout among college students in majors of medical and health-related fields and the moderating effect of gender

**DOI:** 10.1038/s41598-025-29821-6

**Published:** 2025-11-27

**Authors:** Ziao Hu, Dongni Pei, Yu Zhang, Dong Yang, Xihai Gao, Fen Tang, Wen Deng, Yuan Cai, Yurong Yun, Haoyu Zhao, Yedi Zhou

**Affiliations:** 1 Department of Ophthalmology, The Second Xiangya Hospital of Central South University, Changsha, 410011 Hunan China; 2 Department of Liver Surgery, The Second Xiangya Hospital of Central South University, Changsha, 410011 Hunan China; 3 Medical School, Zhangjiajie College, Zhangjiajie, 427000 Hunan China; 4 School of Education, Chuxiong Normal University, Chuxiong, Yunnan, 675099 China; 5 School of Medical Technology, Binzhou Polytechnic College, Binzhou, 256603 Shandong China; 6 Department of Ophthalmology, Autonomous Region & Guangxi Key Laboratory of Eye Health & Guangxi Health Commission Key Laboratory of Ophthalmology and Related Systemic Diseases Artificial Intelligence Screening Technology &Institute of Ophthalmic Diseases, The People’ s Hospital of Guangxi Zhuang, Guangxi Academy of Medical Sciences, Nanning, 530021 China; 7 School of Medicine, Hainan Vocational University of Science and Technology, Haikou, Hainan, 571137 China

**Keywords:** Computer vision syndrome, Academic burnout, Academic stress, Gender, College students in majors of medical and health-related fields, Health care, Health humanities, Psychology, Psychology

## Abstract

College students in majors of medical and health-related fields in the academic environment are at high risk of experiencing academic burnout (AB) and computer vision syndrome (CVS). This study explored the direct relationship between CVS and AB among these students. In addition, the study grounded in Job Demand-resources (JD-R) Theory and Social Role Theory constructed a moderated mediation model to examine the mediating role of academic stress (AS) and the moderating role of gender. A total of 859 college students in majors of medical and health-related fields participated in this cross-sectional study. The results demonstrated that CVS had a significant positive prediction on both AS and AB among these students, AS significantly and positively predicted AB, AS played a significant mediating role in the relationship between CVS and AB. Additionally, gender significantly moderated the prediction of CVS on AB, no significant gender difference in CVS was observed, a significant gender difference in AB was found, with males reporting significantly higher AB than females, once again confirmed in the academic environments that gender is key to understanding burnout. These findings improve our understanding of how CVS contributes to AB and the emergence of gender difference of these students’ AB. Furthermore, to promote gender equality in medical and health-related fields in academic environments, this study provides actionable insights for medical education institutions to mitigate both AB and CVS according to gender differences and the relationship between CVS and AB.

## Introduction

 Since the 21st century, the issue of burnout has received increasing research attention^1^. Burnout is a symptom of mental and physiological exhaustion associated with a profession^2^, resulting from chronic stress associated with one’s profession^3^, and it is prevalent in various fields, particularly among medical and health professionals^4^. The burnout of students in an academic environment is primarily related to academic work^5^, called academic burnout (AB). AB is a multi-dimensional structure that is characterized by variable degrees of emotional exhaustion, depersonalization (indifference to work or study), and lack of personal accomplishment^6^. This issue is also highly prevalent in medical and health-related field education^7^ and may promote negative effects such as dropout intention in the students of these fields^8^. Therefore, the predictive factors of AB among these students in the academic environment should be explored, and action is needed to prevent AB among the students in these fields^7^.

Computer vision syndrome (CVS) is a type of eye physiological issue caused by prolonged use of digital devices with electronic screens^9^. In this digital age, CVS is extremely common among college students in medical and health-related fields^10–12^. Although previous studies have linked physiological health issues to burnout^13–15^, no study has investigated the relationship between ocular physiological health issues and burnout. To fill this gap, this study explored the direct predictive relationship between CVS and academic burnout (AB). We also explored the mediating effect of academic stress (AS) on the relationship between CVS and AB, according to job demand-resources (JD-R) theory, and considered CVS as an extra job demand for the students (demanding them to overcome more difficulties). Previous studies have demonstrated that men and women employ different strategies to cope with risks or health issues^16^. On the basis of this difference, this study hypothesized and explored the moderating role of gender in the relationship between CVS and AB. In addition, from the sociocultural perspective, based on social role theory (SRT), this study also discusses the different social expectations and requirements undertaken by different gender roles, which may be potential reasons for the different strategies used to cope with the risks or diseases of college students of different genders, especially those in medical and health-related fields.

1.1 Computer Vision Syndrome and academic burnout among college students in majors of medical and health-related fields.

The digitization of medical and health fields academic resources is regarded as an important task by the World Health Organization^17^. In medical and health-related domain, academic materials and tasks, such as medical and healthy information^18^, electronic medical records^19^, and medical virtual simulation^20^ should be completed using computers. Computer Vision Syndrome (CVS) is considered a type of physiological symptom caused by prolonged use of digital devices with electronic screens^9^. The American Optometric Association defines CVS as ophthalmologic challenges related to close computer use, such as photophobia of the eyes, burning, blurring of vision, dryness, or itching^21^. CVS has become a global health issue for most computer users^22^ with the advent of the digital era^23^. CVS is highly common among college students in majors of medical and health-related fields in different regions^10–12^. The morbidity rate of these students with CVS was 69.1% in a American study^11^, while in the two Indian studies, it was 77.5%^12^ and 78.6%^10^. This proportion is up to 86% among this type of students in Egyptian^24^.

Previous studies focused on environmental factors more, and pointed out that the critical influencing factors of burnout among students of medical and health-related fields are the environmental factors in learning and working (such as, bad study environment; insufficient support of teachers, staff, and medical colleges; disorganized clinical rotation; and several medical challenges)^7^. However, burnout in the field of medicine is not only related by the environmental factors of study and work, but also by individual health challenges^25^, for instance Psychological health challenges and physiological health challenges. Specifically, previous studies in different countries demonstrated that psychiatric or mental health issues such as sleep disorders^26^, depressive symptoms^27^, addiction^28^ have direct significant impacts on burnout. In addition, physiological health challenges have also been linked to burnout in medical and health professionals, as suggested by some other previous studies^13–15^. A study conducted in Japan found that the higher the number of physiological symptoms (such as fatigue, backache, headache, and stomachache), the greater the burnout scores^15^. Additionally, Davila et al.^14^ surveyed 263 clinicians and identified a significant association of burnout with physiological health issue (pain); however, the mutual relationship was ambiguous. Another study focused on nurses further clarified this relationship by using multiple linear stepwise regression analysis between physiological discomfort factors and burnout, concluding that physiological health issues (back pain severity score) was a positive independent predictor of burnout^13^.

While many prior studies have explored the links between health issues and burnout, the research on the relationship between physiological health problems and burnout is relatively limited. Therefore, it is essential and meaningful to investigate the relationship between physiological health issues and burnout further^29^. Since CVS is a widespread physiological health challenge among students of medical and health-related fields^10–12^, and no previous study has explored the relationship between CVS and these students’ AB, this study considered CVS as a physiological health challenge and examined the potential relationship with CVS on AB in the students of these fields, aiming to fill the research gap. Therefore, this study hypothesized that CVS can predict these students’ AB significantly (H1).

### Hypothesis 1

CVS significantly predicts AB among college students in majors of medical and health-related fields.

### The mediating effect of academic stress (AS)

Stress is a specific relationship between individuals and their environment^30^. When a person is insufficient to cope with their surroundings, individuals are likely to experience stress^30^. Previous studies have shown that physiological problems (such as illness and injury) that disrupt homeostasis can promote stress^31^, and stress has been confirmed as a predictor of burnout^32,33^. Therefore, We suppose that when college students in majors of medical and health-related fields experience CVS in prolonged and intense studies, CVS can predict AS (H2), and AS can predict AB (H3).

Job demand-resources (JD-R) theory divides all working environments into two relatively independent categories to affect people: job demands and job resources. Job demands are defined as the physiological, psychological, social, or organizational aspects of the job that require sustained physical, cognitive, and/or emotional effort and are therefore associated with certain physiological and/or psychological costs^34^. The students in majors of medical and health-related fields are kept in a high-intensity learning environment for a prolonged period because of high expectations^35^, demanding academic requirements, and intense competition in medical and health education institutions^36^. This situation forces them to engage in extended periods of computer use for learning^22^, and widespread cases of CVS among the students of these fields in different regions^10–12,24^. CVS usually may causes inconvenience in the life and studies of students^22^, may reduce the learning their efficiency. According to JD-R theory, in the absence of changes in job resources, these college students may need to spend more physiological and/or psychological costs to meet academic requirements (in other words, job demands increase), which may make it more difficult for them to cope with the academic environment and may promote their AS^31^. The increase in AS may enhance AB of college students in majors of medical and health-related fields, a state of dual mental and physiological exhaustion^2^, owing to long-term stress^3^. Therefore, this study speculates that AS may have a mediating effect in the relationship of CVS on AB (H4).

#### Hypothesis 2

CVS significantly predicts AS among college students in majors of medical and health-related fields.

#### Hypothesis 3

AS significantly predicts AB among college students in majors of medical and health-related fields.

#### Hypothesis 4

AS has a mediating effect on the relationship between CVS and AB among college students in majors of medical and health-related fields.

### The moderating effect of gender

Gender factor has become a hot research topic in recent years^37,38^. Gender was pointed out as a key factor in understanding personal burnout by Fiorilli et al.^39^. The career choice performance between different genders differs^40^, while gender differences in burnout status in many industries exist, including those related to medicine and health^41^. A study conducted among school staff demonstrated that the degree of burnout among men was higher than that among women^42^. However, another study found that the level of burnout among women police officers was higher than that among their male counterparts^43^. Gender difference in burnout was also found among the student group. An Italian study conducted among 494 college students confirmed a significant difference in burnout level among college students of different genders^39^.

However, gender differences have not been reported by most CVS-related studies^44–48^. Still, some studies focus on the relationship between CVS and gender^49,50^. The research conducted by Sawitri and Apriyanti^50^ indicates a significant correlation between gender and CVS. Additionally, Galindo-Romero et al.^49^ found that among individuals with presbyopia aged 45–65 years, the severity of CVS was significantly higher in women compared to men. Therefore, this study hypothesizes that there might be significant gender differences in AB and CVS among the college students in majors of medical and health-related fields (H5).

**Fig. 1:**
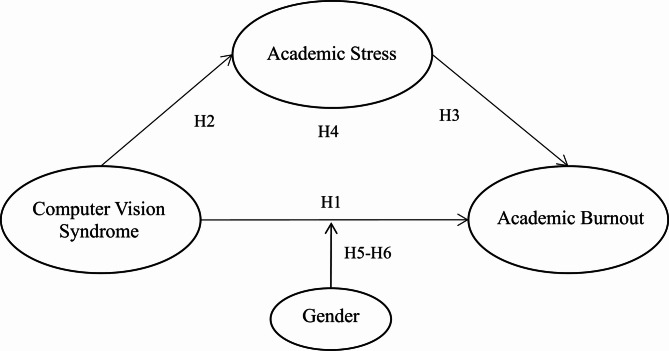
Hypothesized Model.

Previous studies have demonstrated that men and women employ different strategies to cope with risks or diseases^16^. Notably, women appear to utilize coping strategies more effectively than men when dealing with psychological issues^16,51^. A study in the United States during COVID-19 pandemic found that men’s coping mechanisms were more likely to contribute to psychological issues in men during the pandemic, while women tended to adopt (emotion-oriented strategies) and positive reframing to protect women’s mental health and decrease adverse impact of the pandemic^16^. Kristofferzon et al.^51^, after analyzing 41 studies comprehensively, found that to reduce the impact of the disease, women with heart disease could adopt more coping strategies than men. Greenglass et al.^42^ through their empirical study, pointed out that women are better at using coping strategies to reduce burnout than men. Therefore, this study speculates that due to the different strategies men and women use in coping with physiological challenges^16^, gender may have a moderating effect on the relationship of CVS on AB in college students in majors of medical and health-related fields who experience CVS (H6), and contructs a moderated mediation model (Fig. 1).

#### Hypothesis 5

There are significant differences in CVS and AB among college students in majors of medical and health-related fields between different genders.

#### Hypothesis 6

Gender has a moderating effect on the relationship of CVS on AB among college students in majors of medical and health-related fields.

## Method

### Participants and procedures

In order to improve the efficiency of questionnaire collection, we adopted convenience sampling to recruit participants from medical colleges (or schools) at four universities across different regions in China. These medical colleges offer medical and health-related majors such as Clinical Medicine, Medical Technology, and Nursing.

The recruitment posters were set up at the four medical colleges (or schools) by the research team, and the two-dimensional code on the posters could be scanned by the volunteer students to participate in the study. This study was conducted in accordance with the Declaration of Helsinki. The participants were informed regarding the research purpose and confidentiality agreement before filling out the questionnaire. The submission of the questionnaire and data processing was conducted anonymously. If they had any questions while filling out the questionnaire, they could refuse or withdraw at any time. Neither refusal nor withdrawal had any adverse consequences. After being informed and giving consent, participants were allowed to complete the questionnaire using the Questionnaire Star (www.wjx.cn). This study approved by the Ethics Committee of Medical School, Zhangjiajie college (ZJCMS-2025–011).

An a priori power analysis in GPower 3.1 (F test of R^2^ increase; 1 tested predictor [gender × predictor]; total predictors = 4; α = 0.05, two-tailed), assuming a small interaction (f^2^= 0.02), indicated *N* ≈ 398 for 80% power^52,53^. Because gender is the moderator and Chinese college students in medical and health-related field cohorts are often female heavy, we oversampled to preserve power for the interaction and to ensure an adequate male subsample^54^. Therefore, we collected 859 valid responses (238 men, 621 women)^52–54^.

### Instruments

The study variables were assessed using three validated scales—CVS, AS, and AB. All instruments underwent cross-cultural adaptation for Chinese college students. For the Computer Vision Syndrome Questionnaire and the Academic Stress Scale, two bilingual researchers translated English to Chinese. For the Academic Burnout Scale, we used the licensed Chinese version provided by Mind Garden, Inc. An independent bilingual then performed back-translation, after which all translators reached a consensus on the cultural appropriateness of the Chinese versions. Finally, we conducted cognitive interviews with 10 Chinese college students to refine the item clarity. After compiling the three instruments into a single questionnaire, introductory instructions were provided to participants to help them self-report on computer-related symptoms of eye discomfort as well as any study-related stress and burnout. In the introductory instructions, we emphasized anonymity and voluntariness and clarified that there were no right or wrong answers. We used neutral, non-leading instructions and clear wording adapted to the Chinese context to reduce evaluation apprehension and wording-related method variance—procedural remedies recommended to mitigate common method bias (CMB)^55^.

Computer Vision Syndrome. The Computer Vision Syndrome Questionnaire (CVS-Q), developed by del et al.^21^, was used to measure computer-related ocular symptoms, and CVS-Q has been applied in multiple studies targeting Chinese students^56–58^. This questionnaire comprises 16 items rated on a five-point Likert scale, where higher scores indicate more pronounced CVS symptoms. An example item includes “Burning.”

Academic stress. The Perception of Academic Stress Scale (PAS), developed by Bedewy and Gabriel^59^, was used to assess perceived academic stress, this scale was demonstrated to be applicable to Chinese college students by a recent study conducted in 2025^60^. The scale consists of 18 items across the following three dimensions: Academic expectation stress, Faculty work and examination stress, and Students’ academic self-perception stress. Each item is rated on a five-point Likert scale. The higher total scores indicate greater perceived AS.

Academic burnout. The Maslach Burnout Inventory—Student Survey (MBI-SS) was used to gauge learning-related exhaustion. This instrument has already also been used in several Chinese studies to measure the academic burnout level of Chinese college students^61,62^. The MBI-SS comprises 15 items spanning three dimensions—Exhaustion, Cynicism, and Inefficacy, which are scored on a five-point Likert scale. Higher scores reflect heightened AB.

### Data analysis

This study used AMOS 21.0 and SPSS 21.0 for data analysis. AMOS 21.0 was employed for confirmatory factor analysis (CFA), mediation, and moderation analyses, whereas SPSS 21.0 was used for t tests and CMB. The data analysis proceeded in the following three stages:

- Stage 1. CFA was conducted to determine the reliability and validity of each scale. The analysis primarily focused on (1) the overall model fit, item quality (factor loadings and error variances), and inter-factor correlations^63^; (2) the multivariate normality^64,65^; and (3) the internal consistency reliability (as proposed by Nunnally and Bernstein^66^, convergent validity^65^, and discriminant validity (as described by Henseler et al.^67^. Finally, this study assessed CMB via Harman’s single-factor test^55^.
- Stage 2. The structural mediation model was built using items retained from the CFA, and the mediation effects were tested using the bootstrap method^68^.
- Stage 3. Independent-samples t tests were first used to examine gender differences across the variables. Next, a multigroup analysis was conducted to verify potential moderating effects across different groups and to determine if the mediation effect was significant^69^.

### Scale reliability and validity

First, a seven-factor oblique measurement model was specified based on the theoretical dimensions of CVS, AS, and AB, comprising: CVS; Academic expectation stress; Faculty work and examination stress; Students’ academic self-perception stress; Exhaustion; Cynicism; and Inefficacy. During the initial evaluation, six items from the AS factors had factor loadings below 0.50 and were removed (for example, competition with my peers for grades is quite intense). This left a total of 43 valid items for the measurement model. Table 1 presents the factor loadings for the 43 retained items. The criterion that met standardized factor loading ≥ 0.50 included^65^, all items of the CVS (CVS1–CVS16), 12 items of the AS (i.e., Academic expectation stress: AS1–AS3; Faculty work and examination stress: AS4–AS9; Students’ academic self-perception stress: AS10–AS12), and all items of the AB (i.e., Exhaustion: AB1–AB5; Cynicism: AB6–AB9; and Inefficacy: AB10–AB15).

**Table 1:** Questionnaire items and their associated parameter estimates.

No.	Factor loading
CVS1	0.75
CVS2	0.77
CVS3	0.82
CVS4	0.77
CVS5	0.69
CVS6	0.77
CVS7	0.82
CVS8	0.79
CVS9	0.76
CVS10	0.77
CVS11	0.77
CVS12	0.74
CVS13	0.76
CVS14	0.75
CVS15	0.70
CVS16	0.73
AS1	0.71
AS2	0.87
AS3	0.67
AS4	0.73
AS5	0.74
AS6	0.73
AS7	0.75
AS8	0.69
AS9	0.75
AS10	0.75
AS11	0.64
AS12	0.63
AB1	0.80
AB2	0.76
AB3	0.73
AB4	0.86
AB5	0.85
AB6	0.86
AB7	0.85
AB8	0.83
AB9	0.84
AB10	0.82
AB11	0.82
AB12	0.71
AB13	0.63
AB14	0.75
AB15	0.78

Note. For specific content related to CVS1-AB15, please refer to the appendix.

A subsequent analysis of reliability and validity indices indicated that the revised 43-item model achieved a good fit and exhibited sufficient internal consistency and construct validity. First, we examined the measurement model fit and found that the model demonstrated good fit, with all factor loadings greater than 0.50, all error variances significant at *p* < 0.001, and inter-factor correlations indicating meaningful associations. These results are summarized in Table 2. Second, we examined the scales’ internal consistency and model’s construct validity. These results are summarized in Table 3. (1) The data satisfied the normality assumption. The univariate indices met the criteria of skewness |skew| < 3 and kurtosis |kurtosis| < 7, whereas the multivariate kurtosis (Mardia) was lower than p(*p* + 2) = 1935^64,65^. (2) The scales demonstrated internal consistency, with all the Cronbach’s α values ≥ 0.70^66^. (3) The measured variables demonstrated convergent validity. All composite reliability (CR) values are ≥ 0.70^65^; all average variance extracted (AVE) values are ≥ 0.50 or ≥ 0.40 when CR ≥ 0.60^70^. (4) The measured variables demonstrated discriminant validity. Discriminant validity is supported when the 95% confidence interval (CI) of the interfactor correlations excludes 1.00^67^. Finally, we evaluated CMB via Harman’s single-factor procedure via unrotated principal component extraction. The leading component explained 35.14% of the variance—below the 40% heuristic—indicating that CMB is unlikely to be a major concern^55^. This fulfilled the requirements for the following structural model analyses.

**Table 2:** Model fit of measurement model.

Model fit: χ^2^=3315.68, *p*<0.001, χ^2^/df = 3.95, CFI = 0.90, RMSEA = 0.06, SRMR = 0.04
Factor loadings: 0.63–0.86 (all>0.50)
Error variances: 0.21–0.67 (*p*<0.001)
Inter-factor correlations: 0.29–0.88

**Table 3:** Reliability and validity indices.

Univariate normality: Skewness range= [− 0.31, 1.01], Kurtosis range= [− 1.04, 1.00] Multivariate normality: Mardia = 733.87
Cronbach α: CVS = 0.96, AS = 0.87, AB = 0.95.
Convergent validity-CR (CVS = 0.96, AES = 0.80, FWES = 0.87, SASPS = 0.71, E = 0.90, C = 0.91, I = 0.89) and AVE (CVS = 0.58, AES = 0.57, FWES = 0.54, SASPS = 0.46, E = 0.64, C = 0.71, I = 0.57) Discriminant validity: 95% CI=[0.20, 0.91]

CVS = Computer vision syndrome; AS = Academic stress; AB = Academic burnout; AES = Academic expectation stress; FWES = Faculty work and exam stress; SASPS = Students’ academic self-perception stress; E = Exhaustion; C = Cynicism; I = Inefficacy.

## Results

### Mediation test of academic stress (Hypotheses 1–4)

Structural equation modeling demonstrated that CVS significantly and positively predicted AB with a standardized coefficient = 0.46 (*p* < 0.001), accounting for 21% of its variance. After including AS in the model, the direct effect of CVS on AB remained significant but was attenuated (β = 0.24, *p* < 0.01), while CVS also exerted a significant positive effect on AS (β = 0.37, *p* < 0.001). AS, in turn, strongly predicted AB (β = 0.61, *p* < 0.001), and together CVS and AS elucidated 53% of the variance in AB. The significance of the indirect path was confirmed by 5000 bootstrap resamples (indirect effect = 0.23, *p* < 0.01; 95% CI [0.16, 0.29]), indicating that AS partially mediates the relationship between CVS and AB. The overall model fit was acceptable: χ² = 3406.23, *p* < 0.001; χ²/df = 1.70; Comparative fit index (CFI) = 0.90; Root mean square error of approximation (RMSEA) = 0.06; Standardized root mean square residual (SRMR) = 0.05. All item loadings ranged from 0.63 to 0.87, exceeding the 0.50 threshold. Thus, hypotheses 1–4 were supported, with hypothesis 4 confirmed as a partial mediation (Table 4).

**Table 4:** Estimates of the mediation effect between the computer vision syndrome and academic burnout.

Mediating effect	Value	Bias-corrected percentile method 95% confidence interval	
		Lower bound	Upper bound
Total effect	0.47**	0.41	0.53
Direct effect	0.24**	0.17	0.32
Indirect effect	0.23**	0.16	0.29

** *p* < 0.01.

### Gender differences and moderation test (Hypotheses 5–6)

Before exploring whether gender moderated key structural paths, we tested for gender differences in CVS and AB. Male and female students did not show significant differences in CVS scores (males: M = 1.98, standard deviation (SD) = 0.83; females: M = 1.95, SD = 0.75; t = 0.36, *p* > 0.05). In contrast, AB differed by gender, with males reporting higher AB than females (males: M = 2.86, SD = 0.92; females: M = 2.67, SD = 0.77; t = 2.82, *p* < 0.01).

Following this, the sample was divided into male (*n* = 238) and female (*n* = 621) groups, and to test for gender moderation, multigroup structural equation modeling was conducted using the previously established mediation model.

Unconstrained baseline model. An acceptable fit was yielded by allowing all parameters to vary freely across groups: χ^2^ = 4,747.85, *p* < 0.001; χ²/df = 2.79; CFI = 0.88; RMSEA = 0.05; SRMR = 0.06. All factor loadings ranged from 0.58 to 0.88. The direct path from CVS to AB was significant in both groups; however, it was stronger for males (β = 0.35, *p* < 0.001) than that for females (β = 0.18, *p* < 0.001).

Equal-path constrained model. Constraining the CVS→AB path to be equal across genders resulted in χ² = 4,752.79 (with unchanged fit indices). The difference in chi-square relative to the baseline was Δχ^2^(1) = 4.94, *p* < 0.05, indicating the constraint significantly worsened the model fit and thus rejected path equality.

Parameter comparison. The path coefficient difference was confirmed to be statistically significant using a z-test (Z = − 2.20, *p* < 0.05; Table 5), with the male coefficient markedly higher than the female coefficient.

**Table 5:** Test of the moderating effect between the computer vision syndrome and academic burnout.

Parameter			Difference
χ^2^ Value	Unconstrained estimate	4747.85***	4.94*
	Constrained estimate	4752.79***	
Unconstrained Estimate Path Value	Men	0.35***	– 2.20*
	Women	0.18***	

*** *p* < 0.001, * *p* < 0.05.

**Fig. 2:**
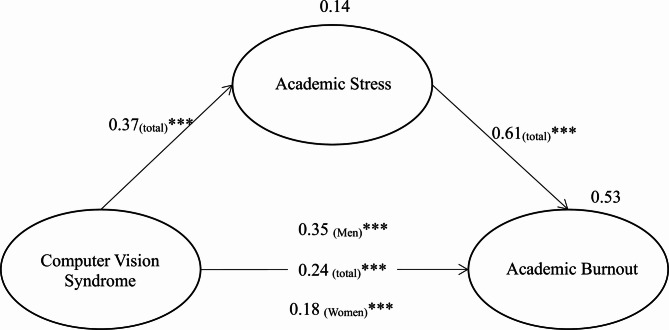
The Mediating and Moderating Model. *p*<0.001***.

To conclude, no gender difference in CVS was noted; however, males demonstrated significantly higher AB than females. Moreover, gender significantly moderated the direct effect of CVS on AB, supporting hypotheses 5 and 6; the hypothetical model of this study was verified (Fig. 2).

## Discussion 

The study results demonstrated that CVS significantly positively predicted the AB for college students in majors of medical and health-related fields, which supported H1 and reconfirmed that burnout is associated with physiological health challenges^14,15,25^, CVS as a physiological health challenge, similar to back pain, can also be used as a predictor of burnout^13^. The burnout is characterized by emotional exhaustion, indifference to work or study, and a low sense of personal accomplishment^6^. These students may experience CVS when they are in a high-intensity learning environment and long-term use of computer learning^21^. Firstly, this physiological health challenge may negatively affect their emotions^71,72^. However, these college students are forced to continue to study intensively owing to heavy academic tasks and lack sufficient time to participate in extracurricular leisure activities^73^ to alleviate CVS. This may promote the prolonged accumulation of negative emotions, may eventually facilitate emotional exhaustion^74^. Secondly, CVS may develop physiological painful experiences such as itching, burning, and dryness^21^. Pain is an unpleasant and aversive experience^75^, and individuals tend to avoid painful experiences^76^. Therefore, their psychological tendency to avoid learning may be related to the painful physiological experiences associated with CVS. This may exhibits indifference and alienation to learning. Additionally, previous studies have demonstrated that visual factors are important predictors of the academic performance of students^77^. When these college students develop CVS, symptoms such as blurred vision can affect their vision^21^, which may impact their academic performance and may promote a lower sense of personal accomplishment. Therefore, CVS presumably significantly positively predicts AB through emotional exhaustion, indifference to work or study, and a low sense of personal accomplishment among these students.

Similar to the previous study^31^, the results of this study demonstrated that CVS positively predicts AS significantly, which supported H2. The study results also verified that AS of college students in majors of medical and health-related fields can significantly positively predict AB, which supported H3. These results were consistent with the findings of the study by Gao^33^, which confirmed that AS is an important predictor of AB. Moreover, this study also indicated that AS has a significant mediating effect on the relationship of CVS on AB, CVS not only directly and positively significantly predicts AB, but also indirectly predicts AB through AS among college students in majors of medical and health-related fields, supporting H4. Specifically, when the students in those fields experience CVS, they may suffer from painful physiological experiences and visual obstacles^21^. This may make it challenging for these students to maintain high learning efficiency. They may have to devote more time and energy to their studies to meet academic requirements. According to JD-R theory, this might imply greater job demands. In the absence of increased job resources, greater job demands may mean greater difficulty for them in coping with the academic environment and thus may promote their academic stress^30^. Moreover, CVS may negatively affect academic performance, may fail to fulfill the expectations of parents, teachers, and themselves^78^, and may also promote their AS^79^. Under long-term AS, AS might positive predict AB^3^, therefore elevation of AS promoted by CVS may advance the level of AB of college students in majors of medical and health-related fields.

The finding of this study that gender is a key factor in understanding academic burnout is in accordance with the study by Fiorilli et al.^39^. Specifically, no significant gender difference of CVS in this study. However, a significant difference in AB between different genders among college students in majors of medical and health-related fields was noted. Males had significantly higher AB than females, similar to the study by Greenglass et al.^42^; therefore, this study partially supported H5. Gender also had a significant moderating effect on the relationship of CVS on AB in this study, and the relationship of CVS on AB was stronger in the male students than that in their female counterparts; thus, supporting H6.

According to social role theory (SRT), the physiological characteristics of men and women are different, and the social roles of men and women may also differ; men are more likely to assume task-oriented (or instrumental) roles, whereas women are more likely to assume socioemotional (or expressive) roles^80^; Barbee et al. pointed out that there are also differences in social requirements for different genders. Gender role expectations may influence whether and how people seek social support. The female role emphasizes nurturance and emotional expressivity, which may make it easier for women to disclose their problems to others, whereas the male role emphasizes achievement, success, strength, and emotional inexpressiveness^81^. Men are encouraged from an early age to be strong, self-reliant and able to address difficulties independently and professionally rather than seeking help, whereas women are allowed to express dependency^81^. Warren pointed out that men expressing sad emotions and seeking help even may encounter severe negative feedback (such as exclusion and punishment)^82^. These different feedback of social roles may make men perceive seeking social support as more threatening than difficult, and women are relatively more likely to seek and receive support and assistance than men from society^81^, so men and women may adopt different strategies to cope with different specific situations^83^, such as physiological problems or risks^16^.

First, based on previous study women are more likely to seek and rely on social help and support to cope with challenges^84^, while men are more likely to avoid them^85^. Therefore, female students experiencing CVS may be more proactive in Seeking help from professionals to diminish the discomfort caused by CVS than their male counterparts. For example, adjustment of the intensity of light based on the situation, along with the screen filters can reduce glare and screen reflection; Intraocular pressure may be relieved by adjusting the distance from the screen, size of the image, and height of the seat; eye tension can be inhibited and work efficiency can be improved by taking short rest, changing the environment, and physical activity; and the use of eye drops or artificial tears can relieve pain owing to dry eyes. The use of corrective eyes can alleviate visual function symptoms, preventing further deterioration^86^. These strategies may lessen the negative impact of CVS, thereby reducing negative emotional reactions^71,72^, thus delay emotional exhaustion in the students. The strategies may reduce physiological pain because of CVS, such as ocular spasm and irritation^21,86^, alleviating the psychological tendency to avoid learning owing to avoiding physiological pain^76^. The aforementioned strategies can also lessen the symptoms of visual function, such as blurred vision^21,86^ to enhance learning efficiency, and may play a protective role in personal accomplishment. In contrast to female roles, male roles are socially required to be emotional inexpressiveness and strong, expose personal problems and difficulties to others, may even be ridiculed by others^81^, may make men are more likely to avoid these problems^85^, hence they are not equivalent to women in alleviating the impact of CVS on AB. In addition, previous studies have shown that social support might play a protective role in reducing the risk of burnout^87^, which may also be a potential reason for the weaker effect of the CVS on academic burnout in female students than in male students, because the social support received by different genders is not the same, and females may receive more social support than males do^81^.

Second, Computers are used for study and work, and can also for recreational activities (such as games). The previous study fund out differences in computer application preferences between genders in the digital era, men use the internet more for entertainment and leisure, while women primarily use it for interpersonal communication and learning^88^. When these students experience CVS, they may suffer physiological pains^21^, possibly resulting in potential negative emotions^71,72^. Male students may more likely to use computer games or other entertainment items to relieve physiological pain when experiencing CVS^89,90^ owing to differences in computer application preferences^88^, and gain pleasure^91^. However, computer games or other related entertainment activities cannot alleviate CVS, and likely even worsen it, probably to further accumulation even aggravation of negative emotions, which may promote a further increase in AB level.

The aforementioned reasons maybe can account for the stronger effect of CVS on burnout in male students than that in female students. This may also explain why the AB of male students is significantly higher than that of their female counterparts while no significant gender difference in CVS is noted.

## Suggestions

At present, CVS is prevalent among college students in majors of medical and health-related fields^10–12,24^; first and foremost, the medical colleges can carry out eye health education to publicize the negative effects of CVS, prompt college students in majors of medical and health-related field pay more attention on CVS, and encourage them check CVS scores regularly, to monitor their own eye health.

Secondly, colleges students of medical and health fields should actively learn and master strategies to alleviate the negative impact of CVS. Specifically, the students of these fields can adjust the light intensity according to their situation and use screen filters to reduce glare and screen reflection. These students can adjust the distance from the screen, the size of the image, and the height of the seat to make their eyes more comfortable. Moreover, they should also pay attention to eye rest and strengthening physical activity to relieve eye tension, and use eye drops or artificial tears to relieve dry eye pain so on.

Third, academic stress also can be regarded as a warning factor for academic burnout. When medical colleges notice that college students of medical and health fields are experiencing CVS, they should promptly pay attention to their academic stress levels and carry out targeted psychological counseling to relieve their academic stress.

Fourth, Medical colleges may consider providing more flexible academic strategies for the students who are experiencing CVS, such as temporarily lowering academic requirements, allowing them to delay submission of homework, providing auxiliary services such as classroom recording, to help them overcome CVS.

Fifth, based on the gender differences discovered in this study, compared with the female students, the male students’ AB is higher predicted by CVS. Therefore, medical colleges should pay more attention to the situation of CVS among male students, publicize the hazards of CVS to male students more, help and urge male students to pay more attention to the prevention and control of CVS.

Sixth, education authorities and institutions help male students to break the traditional gender role thinking. Specifically, encourage male students to actively seek help from the school when they are in difficulties, or provide anonymous support paths to help male students to eliminate their concerns about seeking support.

## Conclusions and limitations

### Conclusions

Overall, according to JD-R Theory and Social Role Theory (SRT), this study explored a few challenges associated with CVS that medical and health-related fields’ college students experience associated with long-term use of computers for academic work in the academic environment. The relationship of CVS on AB in these students, the mediating effect of AS, and the moderating effect of gender were examined. Specifically speaking, CVS can significantly, directly, and positively predict the AB and may indirectly affect the AB through AS in this study. Additionally, gender can moderate the relationship of CVS on AB. The relationship of CVS on AB of male students was stronger than that of their female counterparts, thus and significantly higher AB in male students than that in female students.

This study applied job demand-resources (JD-R) theory to the academic environment and constructed and confirmed that the mediation model of CVS significantly predicts academic burnout in college students in medical and health fields through academic stress. This finding suggests that JD-R theory is applicable not only to the working environment but also to the academic environment and expands the applicable scope of the theory. Moreover, the understanding of job demands (including academic demands in an academic environment) in previous studies may focus more on the work itself, such as the total amount of work and the difficulty of work. In this study, against the background of the digital era, we innovated that the CVS (ocular physiological problem), a hindering factor for personal learning, may also be understood as a type of extra job demand, which further enriched the connotation of job demands in JD-R theory. In addition, on the basis of social role theory, this study confirmed that gender has a significant moderating effect on the relationship between ocular physiological challenge (such as CVS) and academic burnout according to the social requirements and social expectations of different gender roles. We verified and supported that gender is key to understanding burnout again from a sociocultural perspective.

Through a cross-sectional study method, this study revealed that CVS is not only a physiological health challenge but also a measurable and intervenable critical factor for predicting the academic burnout of college students in medical and health-related fields. Our results also revealed that AS had a significant mediating effect on the relationship between CVS and AB, so medical colleges should also include reducing AS in their intervention strategies to alleviate academic burnout among students. Moreover, this study explored the moderating effect of gender on the relationship between the CVS and AB and analyzed the reasons for this moderating effect. The above findings can be used as a practical reference for medical colleges to formulate and implement intervention strategies for alleviating students’ academic burnout.

### Limitations

This study has a few limitations. Firstly, this study was conducted among the Chinese college students in majors of medical and health-related field population, possibly limiting the generalizability of the study’s results. Future studies should consider replicating this study in different countries; thus re-verifying the results in other regions and the general applicability of the conclusions of this study. Secondly, Due to the heavy academic workload of these students, in order to improve the efficiency of questionnaire collection, this study relied on a convenience sample, which may limit generalizability despite our multicohort recruitment, quality control, and sample size adequacy. Future work should use probability or stratified sampling to validate these findings in broader settings and reduce possibly limiting the generalizability of the study’s results. Thirdly, this study followed a cross-sectional design. Future studies should consider conducting longitudinal or experimental studies to re-verify the results of this investigation using multiple research methods.

## Data Availability

The datasets used and/or analyzed during the current study are available from the corresponding author on reasonable request. Permission to use the MBI questionnaire was obtained under a licensed agreement from Mind Garden, Inc.
